# Assessment of diffusion-weighted MRI in predicting response to neoadjuvant chemotherapy in breast cancer patients

**DOI:** 10.1038/s41598-023-27787-x

**Published:** 2023-01-12

**Authors:** Nathalie A. Hottat, Dominique A. Badr, Sophie Lecomte, Tatiana Besse-Hammer, Jacques C. Jani, Mieke M. Cannie

**Affiliations:** 1 Department of Radiology, University Hospital Brugmann, Université Libre de Bruxelles, Place A. Van Gehuchten 4, 1020 Brussels, Belgium; 2 Department of Radiology, UZ Brussel, Vrije Universiteit Brussel, Brussels, Belgium; 3 Department of Obstetrics and Gynecology, University Hospital Brugmann, Université Libre de Bruxelles, Brussels, Belgium; 4 Department of Pathology, University Hospital Brugmann, Université Libre de Bruxelles, Brussels, Belgium; 5 Clinical Research Unit, University Hospital Brugmann, Université Libre de Bruxelles, Brussels, Belgium

**Keywords:** Cancer, Breast cancer

## Abstract

To compare region of interest (ROI)-apparent diffusion coefficient (ADC) on diffusion-weighted imaging (DWI) measurements and Ki-67 proliferation index before and after neoadjuvant chemotherapy (NACT) for breast cancer. 55 women were enrolled in this prospective single-center study, with a final population of 47 women (49 cases of invasive breast cancer). ROI-ADC measurements were obtained on MRI before and after NACT and were compared to histological findings, including the Ki-67 index in the whole study population and in subgroups of “pathologic complete response” (pCR) and non-pCR. Nineteen percent of women experienced pCR. There was a significant inverse correlation between Ki-67 index and ROI-ADC before NACT (r = − 0.443, p = 0.001) and after NACT (r = − 0.614, p < 0.001). The mean Ki-67 index decreased from 45.8% before NACT to 18.0% after NACT (p < 0.001), whereas the mean ROI-ADC increased from 0.883 × 10^–3^ mm^2^/s before NACT to 1.533 × 10^–3^ mm^2^/s after NACT (p < 0.001). The model for the prediction of Ki67 index variations included patient age, hormonal receptor status, human epidermal growth factor receptor 2 status, Scarff-Bloom-Richardson grade 2, and ROI-ADC variations (p = 0.006). After NACT, a significant increase in breast cancer ROI-ADC on diffusion-weighted imaging was observed and a significant decrease in the Ki-67 index was predicted.

Clinical trial registration number: clinicaltrial.gov NCT02798484, date: 14/06/2016.

## Introduction

Neoadjuvant chemotherapy (NACT) is a standard of care for women with locally advanced or inflammatory breast cancer. It is also proposed for downstaging large tumors to allow breast-conserving surgery^1^. Moreover, NACT often including targeted agents is offered to clinically node-negative breast cancer patients with unfavorable tumor profiles especially in HER2-positive and triple negative breast cancer^2^. Immunohistochemical assessment of the proportion of cells stained for the nuclear antigen Ki-67 has become the most used method for measuring breast cancer proliferation. A high level of Ki-67 proliferation index reflects a high tumor aggressiveness and thus a potential chemosensitivity to NACT. Therefore, the ki-67 index is routinely taken into account to select patients for NACT. The Ki-67 index is also considered as a dynamic biomarker of treatment efficacy in samples obtained during and after neoadjuvant therapy^3^.

Nowadays, magnetic resonance imaging (MRI) is the best imaging modality for assessing breast cancer response after NACT and for predicting pathologic response^4–6^. Diffusion-weighted imaging (DWI) reflects the random movement of water molecules in biological tissues and provides quantitative information on tissue cellularity. Tumor lysis, destruction of cell membranes, and increased extracellular space are leading to increased water diffusivity, demonstrating treatment response. A significant increase in the apparent diffusion coefficient (ADC) value is measured after NACT for breast cancer patients, with larger increases in pathologic responders than in non-responders^7,8^. Currently, DWI is widely used for MRI-based monitoring of breast cancer under NACT^9–15^.

A recent study demonstrated that a significant increase in the breast tumor region of interest (ROI) ADC on DWI predicts complete pCR and radiologic responses after one cycle of NACT. The two-dimensional (2D) ROI-ADC measurement of a tumoral target component was accurate and superior to the three-dimensional (3D) whole-lesion-ADC histogram analysis obtained after segmentation of the whole tumor^15^.

The present study aimed to compare the 2D ROI-ADC measurement with the Ki-67 proliferation index before and after NACT to assess ADC changes as a predictive biomarker for changes in the Ki-67 index due to NACT in patients with invasive breast cancer.

## Methods

### Study design and patient selection

This was an analysis of a prospective single-center study conducted between January 2016 and December 2019 on women with invasive breast cancer^15^. This study was approved by the institution’s ethics committee of University Hospital Brugmann (EC 2016/76 B077201628620/I/U) and was also registered at ClinicalTrials.gov (NCT02798484, 14/06/2016). All experiments were performed in accordance with relevant guidelines and regulations. All patients signed informed consent forms and were enrolled according to the following study criteria: Inclusion criteria: (1) female gender, age over 18 years; (2) core needle biopsy-proven invasive breast cancer; (3) indication for NACT; (4) performance status of 0–3 according to the ECOG/WHO/Zubrod score^25^; and (5) evaluable pre-and post-NACT MRI examinations. Exclusion criteria: (i) contraindications to MRI examination; (ii) contraindications to NACT; and (iii) absence of surgery.

**Figure 1:**
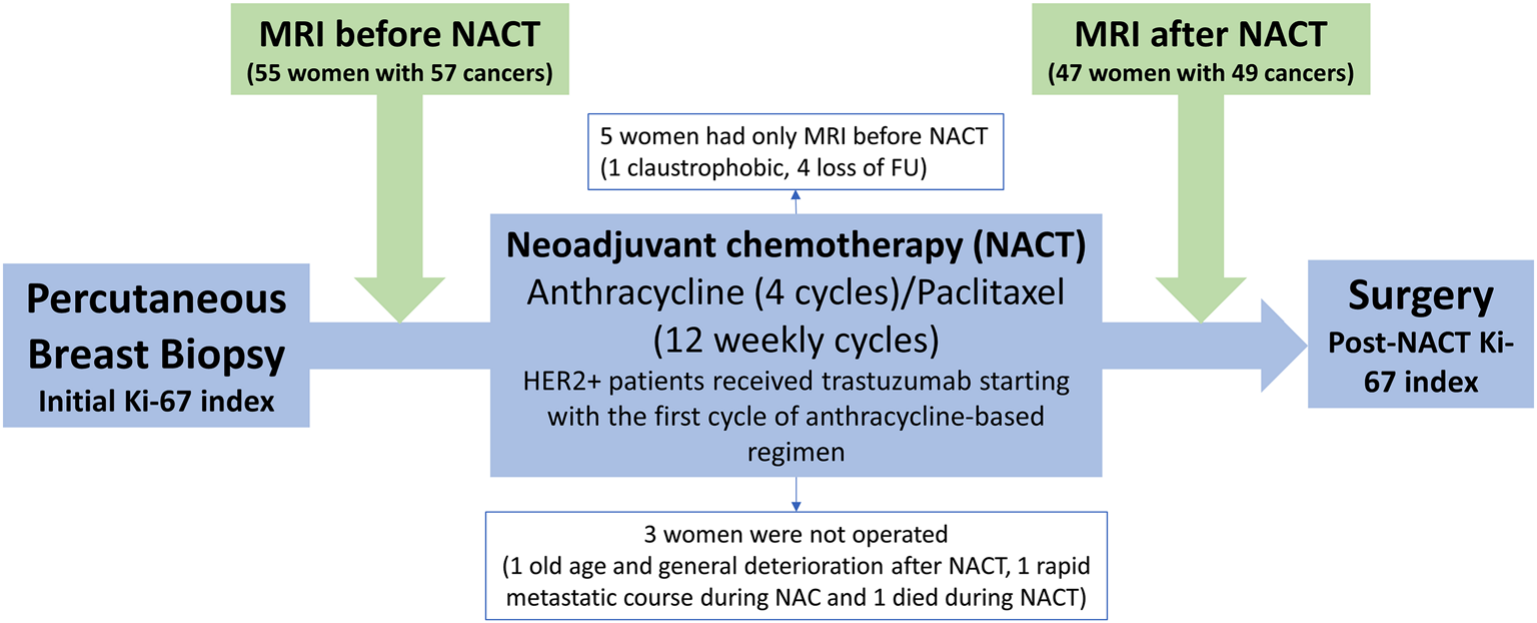
Illustration of the study protocol.

Fifty-five women with 57 invasive breast cancers were included in this study and underwent MRI before starting NACT. Five women were excluded because two of them did not undergo MRI after NACT and three of them did not undergo surgery; thus, the final study sample consisted of 47 women with 49 invasive breast cancers. All patients received the same NACT followed by surgery. NACT consisted of anthracycline-based therapy (4 cycles at intervals of 3–4 weeks), followed by taxane-based therapy (12 weekly cycles of paclitaxel). For patients with HER2-positive tumors (9 patients), trastuzumab was administrated concomitantly with the anthracycline-based regimen (Fig. 1). For each patient, MRI with DCE and DWI before and after the completion of NACT were obtained and analyzed.

### MRI acquisition

MRI examinations were performed on a 3-Tesla system (Philips Ingenia, Best, The Netherlands) using a dedicated breast coil (7-element SENSE) in the prone position. Gadoteric acid (0.2 mmol/kg; Dotarem 0.5 mmol/mL, Guerbet, Aulnay, France) was injected with a power injector (Medrad, Maastricht, The Netherlands). The following sequences were acquired in the axial plane: T2 SPAIR TSE, T1 TSE, diffusion-weighted single-shot echo-planar imaging EPI (b = 0, 50, 400, and 800 s/mm^2^), and dynamic eTHRIVE (3D T1-weighted gradient echo with fat saturation) with gadolinium administration (Supplementary Table S1). The images were sent to a picture archiving and communication system workstation for analysis^15^.

### Image analysis

One radiologist with 15 years of experience in the field of breast MRI interpreted the MRI findings according to the BI-RADS classification^26^. Tumors were identified on DCE subtraction images and localized on DWI before and after treatment. The longest diameter (LD) of the lesion was measured on the DCE subtracted images. The ROI-ADC measurement of the lesion was obtained on the ADC map. These data were acquired prospectively.

The quantitative assessment of DWI consists usually in drawing a region of interest (ROI) on the *b* value image and copying it onto the ADC map, or directly on the darkest part of the ADC map corresponding to the targeted restrictive area on the highest *b* value. The ADC values thus yield quantitative data expressed in the units 10^–3^ mm^2^/s and provide unique quantitative information which can be used for tissue characterization and response assessment. The simplest and common method to quantify the ADC of a tissue is by using summary statistics with a mean ADC value of an ROI. In the present study, a small circular ROI was placed on the darkest part of the lesion on the ADC map focused on the targeted restrictive regions corresponding to the highest signal intensity on the highest b-value, avoiding T2-shine through regions due to necrosis. The size of the ROI was adapted to the size of the signal intensity observed at the highest b value. In the case of multiple targeted components, multiple ROIs (three to five) were used, and the lowest ROI-ADC was retained. In the absence of a targeted restrictive residual tumor, the ROI was placed in the same tissue region as that in the previous examination.

The percentage of change in LD between the two MRI scans (pre-and post-NACT) was calculated as follows: (value before NACT − value after NACT) * 100/value before NACT. Radiologic response was evaluated using the response evaluation criteria in solid tumors (RECIST v1.1) based on the LD changes of the tumor on pre- and post-treatment MRI: complete response (CR) corresponds to the complete resolution of the original imaging finding, partial response (PR) corresponds to an LD decrease of > 30%, progressive disease refers to an LD increase of > 20% or the appearance of new lesions, and stable disease (SD) corresponds to LD interval changes that do not fulfill these three responses^27^.

### Histopathology

The histopathology of the percutaneous core needle biopsy before NACT and that of the surgical specimen after NACT were the reference standards^28,29^. Histological grading was performed according to the modified Scarff-Bloom-Richardson scoring and combined Nottingham classification. Hormone receptor (HR) positivity (estrogen receptor positivity or progesterone receptor positivity) and human epidermal growth factor receptor 2 (HER2) expression were determined from core needle biopsies using immunohistochemistry (IHC). The Ki-67 proliferation index was determined by IHC on the core needle biopsy and surgical specimen (with a Dako MIB-1 antibody). The Ki-67 proliferation index was appraised on 100 cells count on a “hotspot.” Tumors were classified based on the IHC results according to the following molecular subtypes: luminal A (ER-positive, Ki-67 < 20%, and HER2-negative), luminal B (ER-positive with either Ki-67 ≥ 20% or HER2-positive); HER2 enriched (ER-negative and HER2-positive), and triple negative (ER-negative, PR-negative, and HER2-negative). The histology of the surgical specimen was scored according to the 8th edition of the TNM Classification of Malignant Tumors^30^. The pathologic complete response (pCR) reference standard was based on histological analysis of the surgical specimen according to the residual cancer burden (RCB) system^31^. Patients were categorized as having pCR or non-pCR based on the postsurgical histopathological examination findings. pCR was defined as the absence of a residual tumor in the breast and axilla (ypT0 and ypN0) independent of the presence of ductal carcinoma in situ. pCR was evaluated according to the RCB protocol, and data were entered into the RCB calculator of the MD Anderson Cancer Center website (https://www.manderson.org/for-physicians/clinical-tool-resources/clinical-calculators/residual-cancer-burden.html), which automatically calculates the value as follows: RCB-0 = pCR, RCB-I = minimal residual disease, RCB-II = moderate residual disease, and RCB-III = extensive residual disease.

### Statistical analysis

Data were analyzed using SPSS 26 statistical software (IBM SPSS Statistics) and R software version 4.1.2. Continuous variables were expressed as mean ± one standard deviation (SD), while categorical variables were expressed as number (frequency). We used the Shapiro–Wilk test to test the normal distribution of continuous variables. We then used the Mann–Whitney U or Kruskal–Wallis tests to compare the mean values of two or more than two groups, respectively. For comparison of categorical variables, we used either Fisher's exact test or Pearson’s chi-square test, as indicated^32^. Wilcoxon matched-pair signed-rank test was used to compare the median values of the paired samples. Moreover, the Spearman correlation coefficient was calculated to examine the correlation between ROI-ADC and the Ki-67 index. A stepwise backward multiple linear regression that included possible confounding variables (such as, patient age, tumor LD, hormonal receptors status, HER2 status, immunohistochemistry results, SBR grade, and δADC) for the prediction of the δKi67 proliferation index after NACT and a multivariate logistic regression to predict pathologic response were done. ADC and Ki67 proliferation index variations (δ) were calculated as follow: (value before NACT − value after NACT)*100/value before NACT. Statistical significance was assumed when the p-value was < 0.05.

## Results

### Baseline characteristics

The study population included 47 women (median age: 53.4 years, range: 25–84 years) with 49 invasive breast cancer cases (Supplementary Table S2). Among the 49 breast cancers, 32 (65%) were HR-positive/HER2-negative, 8 (16%) were HR-negative/HER2-negative, and 9 (19%) were HR-negative/HER2-positive. Four tumors (8%) were luminal A, 28 were luminal B (57%), and 17 were nonluminal (35%). Twenty-six women (55%) had positive axillary nodes on fine-needle aspiration cytology or core needle biopsy before NACT. Nine women (19%) achieved pCR. Among the nine patients with pCR, six had HER2-positive tumors.

### Variations in Ki-67 index and ROI-ADC according to tumor characteristics

The Ki-67 index increased from 22.5% ± 24.7% in grade 1 tumors to 56.4% ± 25.7% in grade 3 tumors (p < 0.001), whereas ROI-ADC decreased from 1.720 × 10^–3^ mm^2^/s ± 1.177 × 10^–3^ mm^2^/s to 0.799 × 10^–3^ mm^2^/s ± 0.186 × 10^–3^ mm^2^/s (p = 0.026). There was a statistically significant difference in the Ki-67 index values according to the tumor molecular types: luminal A, luminal B, and non-luminal. Nonetheless, the mean ROI-ADC was higher in tumors with the luminal A type than in those with luminal B or non-luminal types, but the difference was not statistically significant (Table 1).

**Table 1:** Baseline tumor characteristics of the study population.

Characteristic	Ki-67 (%)	p-value	ROI-ADC (× 10^–3^ mm^2^/s)	p-value
Grade		0.001		0.026
1	22.5 ± 24.7		1.720 ± 1.177	
2	29.9 ± 15.9		0.934 ± 0.147	
3	56.4 ± 25.7		0.799 ± 0.186	
Molecular subtypes		0.005		0.096
HR−/HER2−	69.4 ± 24.7		0.712 ± 0.179	
HR+/HER2−	37.0 ± 21.5		0.930 ± 0.340	
HR−/HER2+	56.2 ± 27.6		0.871 ± 0.189	
Luminal A	8.8 ± 2.5	< 0.001	1.369 ± 0.821	0.176
Luminal B	41.1 ± 19.9		0.867 ± 0.158	
Non-luminal	62.4 ± 26.3		0.796 ± 0.196	

*HER2* human epidermal growth factor receptor 2, *HR* hormone receptor, *Ki-67 Ki-67* proliferation index, *ROI* region of interest, *ADC* apparent diffusion coefficient.

### Correlation between Ki-67 index and ROI-ADC before and after NACT

Table 2 presents the tumor characteristics on histology and MRI before and after NACT. There was a statistically significant decrease of Ki-67 index and LD (p < 0.001), whereas ROI ADC significantly increased. Before NACT, there was a significant inverse correlation between Ki-67 index and ROI-ADC (r = − 0.443, p = 0.001) (Supplementary Fig. S1). A similar finding was noted after NACT (r = − 0.614, p < 0.001) (Supplementary Fig. S2). The model for the prediction of δki67 proliferation index according to the stepwise backward multiple linear regression included patient age, HR status, HER2 status, SBR grade 2, and δADC (p = 0.006) (Supplementary Table S3).

**Table 2:** Tumor characteristics on histology and MRI before and after NACT.

Characteristic	Before NACT N = 49	After NACT N = 49	p-value
Histology			
SBR			
0	0	12 (24.5%)	–
1	2 (4.1%)	5 (10.2%)	
2	17 (34.7%)	19 (38.8%)	
3	30 (61.2%)	13 (26.5%)	
Ki-67, %	45.8 ± 26.1	18.0 ± 26.1	< 0.001
Tumor diameter, mm	–	22.0 ± 22.4	–
MRI			
LD, mm	50.0 ± 27.8	22.6 ± 22.7	< 0.001
LD DWI, mm	46.7 ± 27.8	22.8 ± 24.2	< 0.001
ROI-ADC, × 10^–3^ mm^2^/s	0.883 ± 0.302	1.533 ± 0.551	< 0.001

*SBR* Scarff-Bloom-Richardson grade, *Ki-67* Ki-67 proliferation index, *LD* largest diameter, *ROI* region of interest, *ADC* apparent diffusion coefficient, *NACT* neoadjuvant chemotherapy, *DWI* diffusion-weighted imaging, *MRI* magnetic resonance imaging.

### Variations in Ki-67 index and ROI-ADC according to pathologic response

**Figure 2:**
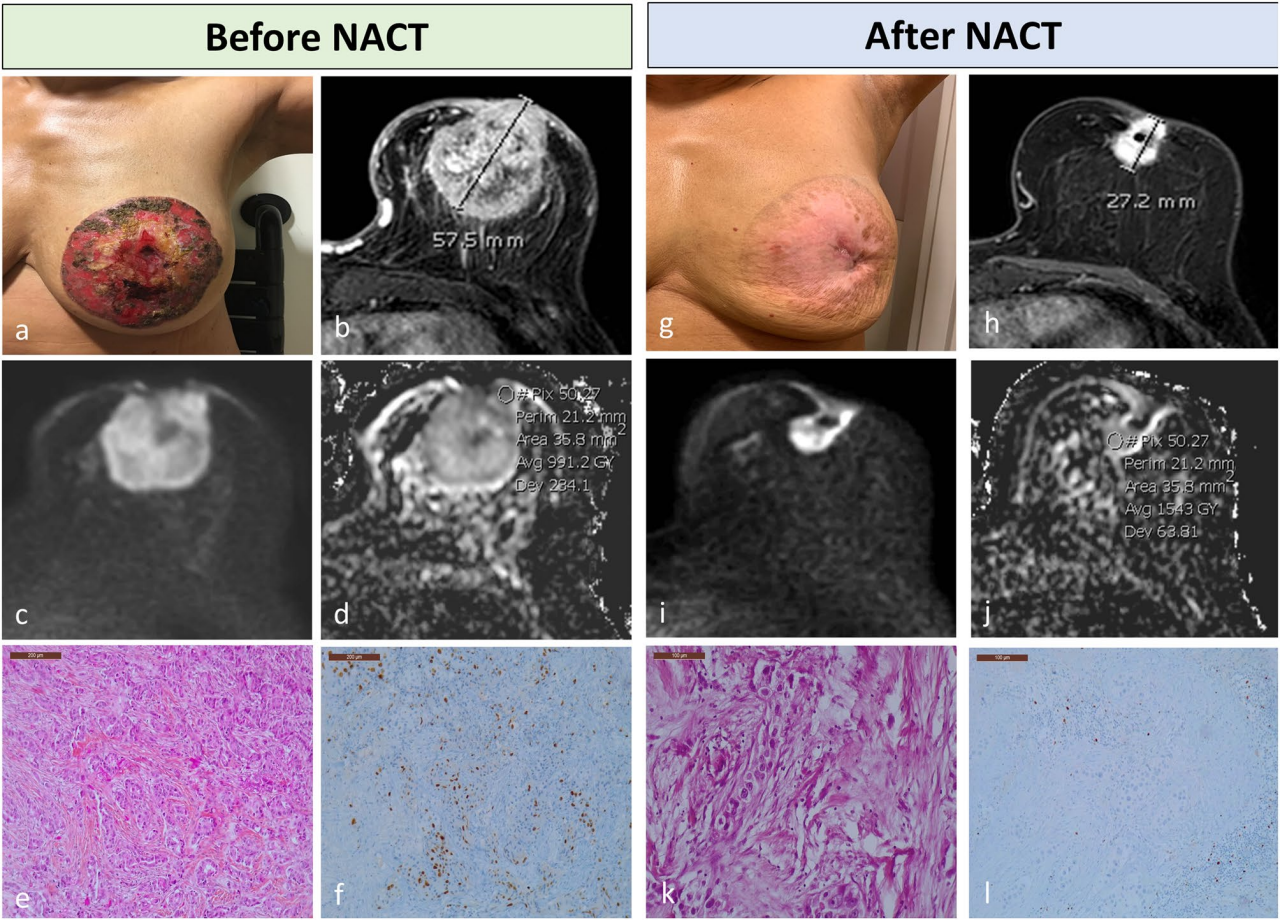
Images of a 61-year-old woman who received neoadjuvant chemotherapy (NACT) for grade 3 hormone receptor–negative/human epidermal growth factor receptor–positive cancer of the left breast. The patient had residual disease at surgery and therefore did not experience pCR (RCB class: II). Before NACT: physical examination findings showing a large skin ulceration (cT4bN1) (**a**), axial DCE T1-WI showing a large, irregular, and heterogeneous mass of size 5.75 cm in LD (**b**), axial DWI showing a high signal intensity mass at *b* = 800 (**c**) and an ROI-ADC value of 0.991 × 10^–3^ mm^2^/s placed on the darkest part of the ADC map (**d**), HE staining of core needle biopsy showing an IDC SBR3 (**e**). IHC of the core needle biopsy showing nuclear positivity for a Ki-67 proliferation index of 30% (**f**). After NACT: cicatrization of the skin ulceration (ycT2N0) (**g**), axial DCE T1-WI showing a residual mass of size 2.72 cm in LD corresponding to a partial response (decrease of 52.7% in LD) (**h**), axial DWI showing a residual high-signal-intensity mass at *b* = 800 (**i**), and an ROI-ADC value of 1.543 × 10^–3^ mm^2^/s placed on the darkest part of the ADC map avoiding clip artifacts (increase of 55.7% in ROI-ADC) (**j**), HE staining of the surgical specimen showing a residual IDC SBR2 of size 2.2 cm (ypT2N1_(mi)_, RCB-II) (**k**), and IHC showing nuclear positivity for a residual Ki-67 proliferation index of 5% (**l**). *ROI* region of interest, *ADC* apparent diffusion coefficient, *pCR* pathologic response, *RCB* residual cancer burden, *DCE* dynamic contrast enhanced, *WI* weighted imaging, *LD* largest diameter, *DWI* diffusion-weighted imaging, *HE* hematoxylin and eosin, *IDC* invasive ductal carcinoma, *IHC* immunohistochemistry.

**Figure 3:**
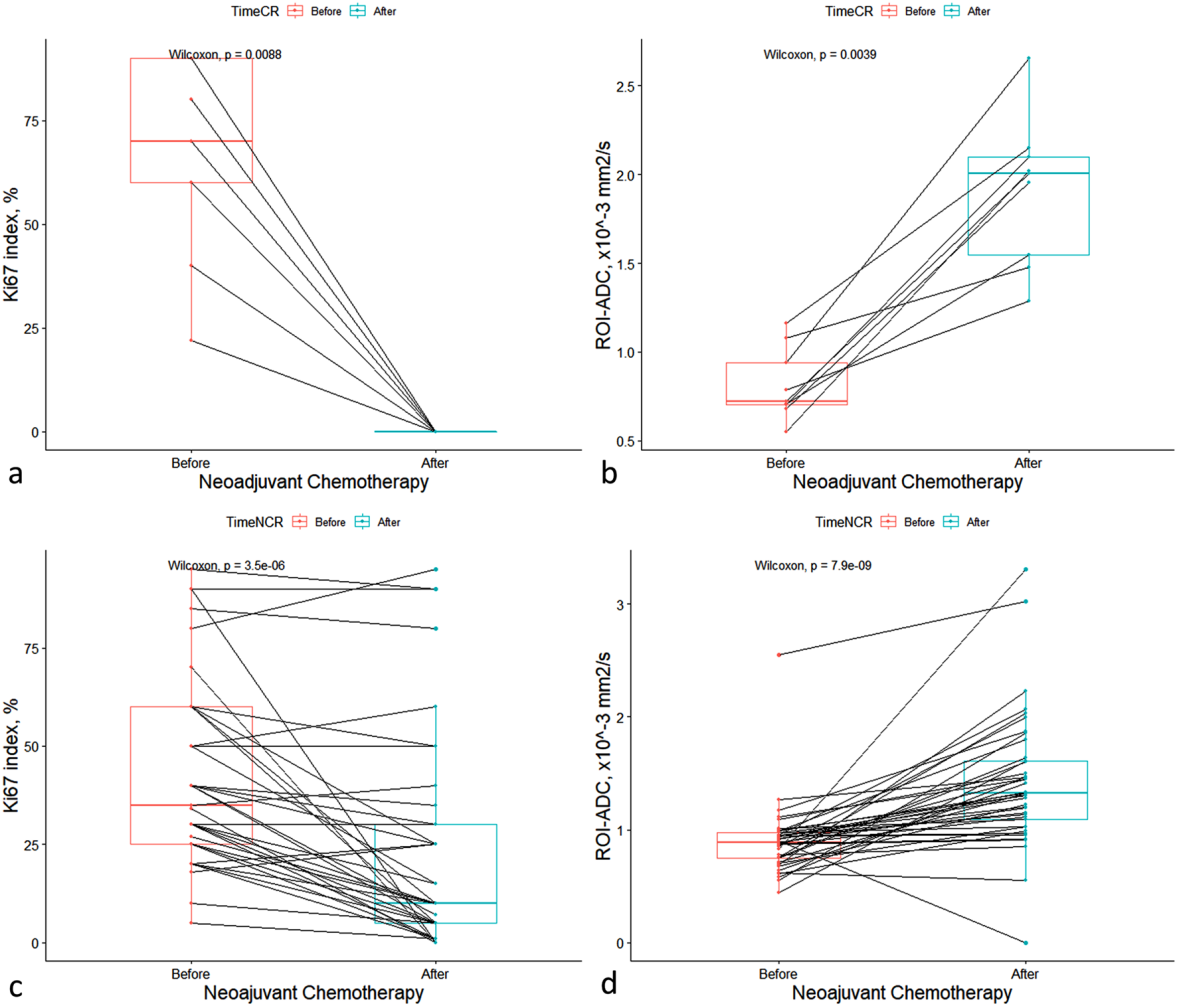
Changes in the ROI-ADC and Ki-67 index before and after neoadjuvant chemotherapy (NACT) in pathologic complete response (pCR) (**a**,**b**) and non-pCR (**c**,**d**) patients. *ROI* region of interest, *ADC* apparent diffusion coefficient.

After NACT, the Ki-67 index of tumors with pCR decreased to 0%, whereas the Ki-67 index of tumors without pCR decreased to 22.1% ± 27.3% (p < 0.001). The ROI-ADC of tumors with pCR increased to 1.910 × 10^–3^ mm^2^/s ± 0.415 × 10^–3^ mm^2^/s whereas those with non-pCR increased only to 1.448 × 10^–3^ mm^2^/s ± 0.546 × 10^–3^ mm^2^/s (p = 0.004) (Table 3). There was an inverse correlation between Ki-67 index and ROI-ADC in the pCR and non-pCR groups after NACT. However, the variation in the pCR group was more pronounced (Fig. 2). The variations in LD on DCE, ROI ADC, and Ki-67 index before and after NACT decreased significantly from RCB class 0 to RCB class 3 (Table 4, Fig. 3). ADC variation before and after NACT was predictive for pCR (Supplementary Table S4).

**Table 3:** Tumor characteristics of the study population according to pathologic complete response.

Characteristic	pCR N = 9	Non-pCR N = 40	p-value
Tumor histology			0.791
Invasive ductal carcinoma	9 (100%)	38 (95.0%)	
Invasive lobular carcinoma	0	1 (2.5%)	
Others	0	1 (2.5%)	
Grade			0.166
1	0	2 (5.0%)	
2	1 (11.1%)	16 (40.0%)	
3	8 (88.9%)	22 (55.0%)	
Molecular subtypes			< 0.001
HR−/HER2−	1 (11.1%)	7 (17.5%)	
HR+/HER2−	2 (22.2%)	30 (75.0%)	
HR−/HER2+	6 (66.7%)	3 (7.5%)	
Luminal A	0	4 (10.0%)	0.010
Luminal B	2 (22.2%)	26 (65.0%)	
Non-luminal	7 (77.8%)	10 (25.0%)	
Before NACT			
Ki-67, %	66.9 ± 24.0	41.1 ± 24.3	0.009
ROI-ADC on DWI, × 10^–3^ mm^2^/s	0.814 ± 0.202	0.899 ± 0.321	0.379
LD DCE MRI, mm	50.1 ± 34.0	50.0 ± 26.7	0.713
LD DWI, mm	50.1 ± 35.2	45.9 ± 26.3	0.970
After NACT			
Ki-67, %	0	22.1 ± 27.3	< 0.001
ROI-ADC on DWI, × 10^–3^ mm^2^/s	1.910 ± 0.415	1.448 ± 0.546	0.004
LD DCE MRI, mm	0.8 ± 2.3	27.6 ± 22.3	< 0.001
LD DWI, mm	0	27.9 ± 24.0	< 0.001

*HER2* human epidermal growth factor receptor 2, *HR* hormone receptor, *pCR* pathologic complete response, *Ki-67* Ki-67 proliferation index, *NACT* neoadjuvant chemotherapy, *ROI* region of interest, *ADC* apparent diffusion coefficient, *LD* largest diameter, *DCE-MRI* dynamic contrast-enhanced magnetic resonance imaging, *DWI* diffusion-weighted imaging.

**Table 4:** Variations in LD and their effect on DCE MRI, ROI-ADC, and Ki-67 index after NACT according to radiologic response and RCB class.

	LD decrease, %	ROI-ADC increase, %	Ki-67 decrease, %
RCB class			
0	100.0 ± 0	146.5 ± 74.7	100.0 ± 0
I	68.2 ± 41.2	126.4 ± 93.6	82.9 ± 27.7
II	42.0 ± 30.5	64.0 ± 71.0	39.8 ± 43.2
III	16.8 ± 15.0	28.7 ± 34.1	42.6 ± 37.8
p-value	< 0.001	0.002	< 0.001
Radiologic response			
CR	100.0 ± 0	148.2 ± 70.4	100.0 ± 0
PR	53.4 ± 28.8	78.1 ± 75.4	49.5 ± 42.8
SD	12.0 ± 15.6	41.6 ± 71.3	39.3 ± 39.9
p-value	< 0.001	0.001	< 0.001

*ADC* apparent diffusion coefficient, *CR* complete response, *DCE MRI* dynamic contrast-enhanced magnetic resonance imaging, *Ki-67* Ki-67 proliferation index, *LD* largest diameter, *NACT* neoadjuvant chemotherapy, *PR* partial response, *RCB* residual cancer burden, *RECIST* response evaluation criteria in solid tumors, used for evaluating radiologic response; *ROI* region of interest; *SD* stable disease.

### Variations in Ki-67 index and ROI-ADC according to radiologic response

According to the RECIST criteria, the decrease in LD on DCE and the Ki-67 index after NACT was significantly lower in lesions with SD than in lesions with PR or CR, similar to the increase in ROI-ADC (Table 4). No progressive disease was observed in the final study population.

## Discussion

The present study compared the ROI-ADC measurement of a targeted tumor component and the Ki-67 proliferation index before and after NACT to assess the tumoral response of invasive breast cancer. The results of this study showed an inverse correlation between ROI-ADC and Ki-67 proliferation index after NAC in patients with breast cancer. This correlation remained valid after grouping the tumors according to their pCR or radiologic response.

The Ki-67 proliferation index is a prognostic marker based on the molecular subtype of breast cancer and is increasingly used to assess and manage breast cancer^3,16^. In fact, changes in the Ki-67 proliferation index due to NACT can independently predict the prognosis of patients with breast cancer^17,18^. The Ki-67 proliferation index measures the percentage of tumor cells that are positive for Ki-67 staining; the more positive cells there are, the more quickly they divide and form new cells, reflecting the aggressiveness of the tumor. In breast tumors, an index of more than 20% is considered high.

Some studies have analyzed the association between ADC measurements and the Ki-67 proliferation index in breast cancers, with contradictory results. In 2015, Molinari et al. showed a correlation between lower ADC values and a higher Ki-67 proliferation index in a population of 115 patients with MRI examinations performed according to the same DWI protocol at 1.5 T^19^. This result is consistent with that reported by Mori et al. in 2015 in a population of 86 patients with luminal-type invasive breast cancers, suggesting that the mean ROI-ADC of tumors extracted from the same DWI protocol would be useful for estimating the Ki-67 index^20^. In 2018, Surov et al. reported in a multicenter study that ADC cannot be used as a surrogate marker for proliferation activity and/or for tumor grade in a population of 845 breast cancer patients, including a large variety of breast tumor histology types, including noninvasive breast cancers with a mean ADC of 0.980 and a mean Ki-67 of 12% from six centers with different scanners (different strength magnetic fields, 1.5 and 3 T and different vendors) and different b-values^21^. However, in our monocentric study population of invasive breast cancers selected for NACT, there was a correlation between a higher Ki-67 index and a lower ADC with regard to the histological grade of the tumor. Interestingly, the mean Ki-67 index was 46%, and the mean ROI-ADC value was 0.888 × 10^–3^ mm^2^/s with a mean LD of 50 mm, which corresponds to very low-to-low ADC values considering the ADC ranges based on recent meta-analyses evaluating DWI in differentiating benign and malignant lesions^22,23^.

While some studies have reported correlations between ADC values and Ki-67 index in breast cancers, only very few data have been published on ADC values and Ki-67 index changes after NACT. Nevertheless, ADC and Ki-67 proliferation indices are both tumor markers. In the present study, the mean Ki-67 index before NACT was higher in patients who achieved pCR than in those who did not. There was no significant difference in the ROI-ADC values before NAC between pCR and non-pCR patients. Moreover, the results of the present study showed an inverse correlation between ROI-ADC and Ki-67 proliferation index after NAC in patients with breast cancer. This correlation remained valid after grouping the tumors according to their pCR or radiologic responses. Our results are consistent with the most comparable study published in 2019 by Luo et al., suggesting that comparison of pre- and post-NACT ADC values can be used to estimate the change in Ki-67 index in a retrospective study that included 87 patients with invasive breast cancer and showed a negative correlation between the change in ADC values and the Ki-67 index due to NACT^24^. The authors suggested that changes in ADC values might be used as a surrogate marker for changes in the Ki-67 index in the NACT response of patients with invasive breast cancer. Because the authors did not consider the radiologic response or the patients who achieved pCR, the comparison with our study was limited.

This study had some limitations. First, only one radiologist performed ROI-ADC measurements. Second, only one pathologist assessed the Ki-67 proliferation index. However, the pathologist followed the recommendations of the international Ki-67 in breast cancer working published to minimize the variation in analytical practice and to standardize the methodology^3^. Our study has several strengths. All patients underwent two consecutive MRI examinations before and after the same NACT protocol. All MRI examinations were standardized and performed prospectively using the same 3-T magnet. The post-processing was standardized. This study demonstrated that ADC measurement is a powerful functional parameter that offers information on tumor cellularity and could provide reliable, fast, noninvasive, and inexpensive prediction of breast cancer response to NACT. DWI does not require contrast agent injection and lasts for only a few minutes. After NACT, a significant increase in breast cancer ADC value on DWI was observed, which predicted a significant decrease in the Ki-67 index in patients with radiological response and in patients with pCR. Therefore, we recommend the systematic use of an ROI targeted at the lowest breast cancer ADC in patients before and after NACT.

The results of this study demonstrate that an inverse correlation between ROI-ADC and Ki-67 proliferation index is observed after NACT in breast cancer patients with pCR and patients with radiologic response. This finding could validate the reliability of the ADC value as a routine biomarker in assessing breast cancer response to NACT. Further studies with larger cohorts of patients should be realized to confirm our results.

## Supplementary Information

Supplementary Information.

## Data Availability

The datasets generated during and/or analysed during the current study are available from the corresponding author on reasonable request.
