# Supplementary material for: Assessment of diffusion-weighted MRI in predicting response to neoadjuvant chemotherapy in breast cancer patients
Source: Sci Rep. 2023 Jan 12;13:614. doi: 10.1038/s41598-023-27787-x (PMC9837175; doi:10.1038/s41598-023-27787-x)
Supplement: Supplementary file 1 — Supplementary Information. [file 41598_2023_27787_MOESM1_ESM.docx]

**Assessment of diffusion-weighted MRI in predicting response to neoadjuvant chemotherapy in breast cancer patients**

Nathalie A. Hottat MD, Dominique A. Badr MD, Sophie Lecomte MD, Tatiana Besse-Hammer MD, PhD, Jacques C. Jani MD, PhD and Mieke M. Cannie MD, PhD.

**Supplementary material**

**Supplementary table 1**: 3-T MRI acquisition parameters.

| **Weighting** | **T2** | **T1** | **Diffusion** | **Dyn 3D T1 fs** |
| --- | --- | --- | --- | --- |
| Sequence name | TSE | TSE | DWI | eTHRIVE |
| Acq. Nr of slices | 60 | 60 | 70 | 80 |
| Acq. slice thickness/gap (mm) | 3/0 | 3/0 | 3/0 | 4/0 |
| Field of view (read × phase) | 320 × 339 | 320 × 339 | 280 × 378 | 320 × 320 |
| Acq. matrix (read × phase) | 320 × 279 | 400 × 330 | 128 × 172 | 292 × 292 |
| Phase encoding direction | LR | LR | LR | LR |
| SENSE factor | 2 | 2 | 4 | 2 × 1 |
| Phase oversampling (mm) | - | - | 2 × 62 | - |
| TR (msec)/TE (msec) | 5000/60 | 400/10 | 12576/74.4 | 4.6/2.2 |
| Turbo factor | 14 | 5 | 59 | 40 |
| Flip angle | 90 | 90 | 90 | 10 |
| Fat suppression | SPAIR | - | SPAIR | SPAIR |
| b-values (s/mm^2^) | - | - | 0, 50, 400, 800 | - |
| Acq. duration (min:sec) | 02:45 | 03:27 | 05:39 | 00:52 × 8 |

Abbreviations: Acq, acquisition; Nr, number; TR, repetition time; TE, echo time; T2, T2-weighted; T1, T1-weighted; Dyn 3D T1 fs: dynamic three-dimensional T1-weighted with fat suppression; TSE, turbo spin echo; DWI, diffusion-weighted imaging; LR, left-right; SENSE, sensitivity encoding; SPAIR, spectral attenuation inversion recovery; 3-T MRI, 3-Tesla magnetic resonance imaging.

**Supplementary table 2**: Baseline characteristics of the study population.

| Patients | Age | Tumor type | SBR (1,2,3) | Luminal type (A,B,0) | HR+ | HER2+ | TN | Ki-67 (%) | ax. LN |
| --- | --- | --- | --- | --- | --- | --- | --- | --- | --- |
| 1 | 48.93 | IDC | 3 | B | Yes | No | No | 80 | No |
| 2 | 59.61 | IDC | 3 | B | Yes | No | No | 40 | Yes |
| 3 | 41.04 | IDC | 3 | 0 | No | Yes | No | 60 | Yes |
| 4 | 35.02 | IDC | 2 | B | Yes | No | No | 60 | Yes |
| 5 | 68.98 | IDC | 3 | 0 | No | No | Yes | 85 | No |
| 6 | 77.48 | IDC | 3 | 0 | No | No | Yes | 25 | No |
| 7 | 64.52 | IDC | 3 | B | Yes | No | No | 60 | No |
| 8 | 70.69 | IDC | 3 | 0 | No | No | Yes | 90 | No |
| 9 | 38.49 | IDC | 3 | B | Yes | No | No | 30 | Yes |
| 10 | 51.79 | IDC | 3 | B | Yes | No | No | 40 | No |
| 11 | 53.99 | IDC | 3 | B | Yes | No | No | 40 | Yes |
| 12 | 53.54 | IDC | 3 | B | Yes | No | No | 35 | Yes |
| 13 | 25.00 | IDC | 2 | B | Yes | No | No | 30 | Yes |
| 14 | 47.53 | IDC | 3 | 0 | No | Yes | No | 90 | No |
| 15 | 56.97 | IDC | 3 | B | Yes | No | No | 60 | Yes |
| 16 | 50.91 | IDC | 3 | 0 | No | Yes | No | 40 | Yes |
| 17 | 76.16 | IDC | 3 | 0 | No | No | Yes | 40 | No |
| 18 | 67.88 | IDC | 2 | A | Yes | No | No | 10 | No |
| 19 | 42.72 | IDC | 2 | B | Yes | No | No | 20 | Yes |
| 20 | 64.01 | IDC | 2 | B | Yes | No | No | 50 | Yes |
| 21 | 36.88 | IDC | 2 | B | Yes | No | No | 20 | Yes |
| 22 | 52.60 | IDC | 3 | B | Yes | No | No | 90 | Yes |
| 23 | 84.14 | IDC | 2 | B | Yes | No | No | 20 | No |
| 24 | 45.57 | IDC | 3 | 0 | No | No | Yes | 80 | Yes |
| 25 | 73.49 | IDC | 1 | B | Yes | No | No | 40 | Yes |
| 26 | 44.52 | IDC | 3 | 0 | No | No | Yes | 95 | Yes |
| 27 | 33.68 | IDC | 2 | B | Yes | No | No | 18 | No |
| 28 | 46.24 | IDC | 3 | 0 | No | No | Yes | 70 | No |
| 29 | 52.93 | IDC | 3 | 0 | No | Yes | No | 90 | No |
| 30 | 46.40 | IDC | 3 | 0 | No | Yes | No | 50 | Yes |
| 31 | 45.98 | IDC | 3 | B | Yes | No | No | 70 | Yes |
| 32 | 54.96 | IDC | 3 | B | Yes | No | No | 20 | No |
| 33 | 71.41 | ILC | 2 | B | Yes | No | No | 20 | No |
| 33 | 71.41 | IDC | 3 | B | Yes | No | No | 27 | No |
| 34 | 61.17 | IDC | 3 | 0 | No | Yes | No | 90 | No |
| 35 | 51.73 | IDC | 2 | B | Yes | No | No | 25 | Yes |
| 36 | 79.35 | IDC | 3 | B | Yes | No | No | 25 | No |
| 37 | 74.63 | IDC | 2 | A | Yes | No | No | 10 | No |
| 37 | 74.63 | IDC | 2 | B | Yes | No | No | 30 | Yes |
| 38 | 66.50 | IDC | 2 | B | Yes | No | No | 50 | No |
| 39 | 53.37 | IMC | 1 | A | Yes | No | No | 5 | Yes |
| 40 | 30.67 | IDC | 3 | B | Yes | No | No | 60 | Yes |
| 41 | 61.55 | IDC | 3 | 0 | No | Yes | No | 30 | Yes |
| 42 | 33.74 | IDC | 3 | A | Yes | No | No | 10 | Yes |
| 43 | 44.32 | IDC | 2 | B | Yes | No | No | 30 | Yes |
| 44 | 59.94 | IDC | 3 | 0 | No | No | Yes | 70 | No |
| 45 | 62.90 | IDC | 2 | 0 | No | Yes | No | 22 | Yes |
| 46 | 46.84 | IDC | 2 | 0 | No | Yes | No | 34 | Yes |
| 47 | 57.09 | IDC | 2 | B | Yes | No | No | 60 | No |

Abbreviations: Ax. LN, axillary lymph node; HER2, human epidermal growth factor receptor 2; HR, hormonal receptor; IDC, invasive ductal carcinoma; ILC, invasive lobular carcinoma; IMC, invasive mucinous carcinoma; SBR, Scarff-Bloom-Richardson grade; TN, triple negative.

**Supplementary table 3:** Stepwise backward multiple linear regression for the prediction of the variation of the ki67 proliferation index pre and post neoadjuvant chemotherapy.

|  | **Estimate** | **Std. Error** | **T value** | **P-value** |
| --- | --- | --- | --- | --- |
| **Intercept** | -23.93 | 29.75 | -0.80 | 0.426 |
| **Age** | 0.64 | 0.40 | 1.60 | 0.116 |
| **HR** | 45.94 | 16.42 | 2.80 | 0.008 |
| **HER2** | 59.29 | 18.89 | 3.14 | 0.003 |
| **Grade 2 (SBR)** | -23.63 | 12.24 | -1.93 | 0.060 |
| **ADC variations** | -0.18 | 0.07 | -2.51 | 0.016 |

Residual standard error: 37.74; degree of freedom: 43; Multiple R-squared: 0.3089; Adjusted R-squared: 0.2285; F-statistic: 3.843, p-value = 0.006.

Abbreviations: ADC: apparent diffusion coefficient; HER2: human epidermal growth factor receptor 2; HR: hormonal receptor; Ki-67: Ki-67 proliferation index; SBR: Scarff-Bloom-Richardson grade; std. error: standard error.

**Supplementary table 4:** Multivariate logistic regression for the prediction of the variation of pathologic response.

| Variables | Adjusted OR | 95% confidence interval | p-value |
| --- | --- | --- | --- |
| Grade 1 | Reference |  |  |
| Grade 2 | - | - | 0.999 |
| Grade 3 | - | - | 0.999 |
| HR-/HER2- (TN) | Reference |  |  |
| HR+/HER2- (HR) | - | - | 0.999 |
| HR-/HER2+ (HER2) | 0.031 | 0.001-1.270 | 0.067 |
| Luminal A | Reference |  |  |
| Luminal B | - | - | 0.999 |
| ADC before NACT | 0.994 | 0.984-1.003 | 0.191 |
| ADC variations | 0.975 | 0.951-0.999 | 0.043 |

Abbreviations: ADC: apparent diffusion coefficient; HER2: human epidermal growth factor receptor 2; HR: hormonal receptor; Ki-67: Ki-67 proliferation index; NACT: neoadjuvant chemotherapy; SBR: Scarff-Bloom-Richardson grade; std. error: standard error; TN: triple negative.

**
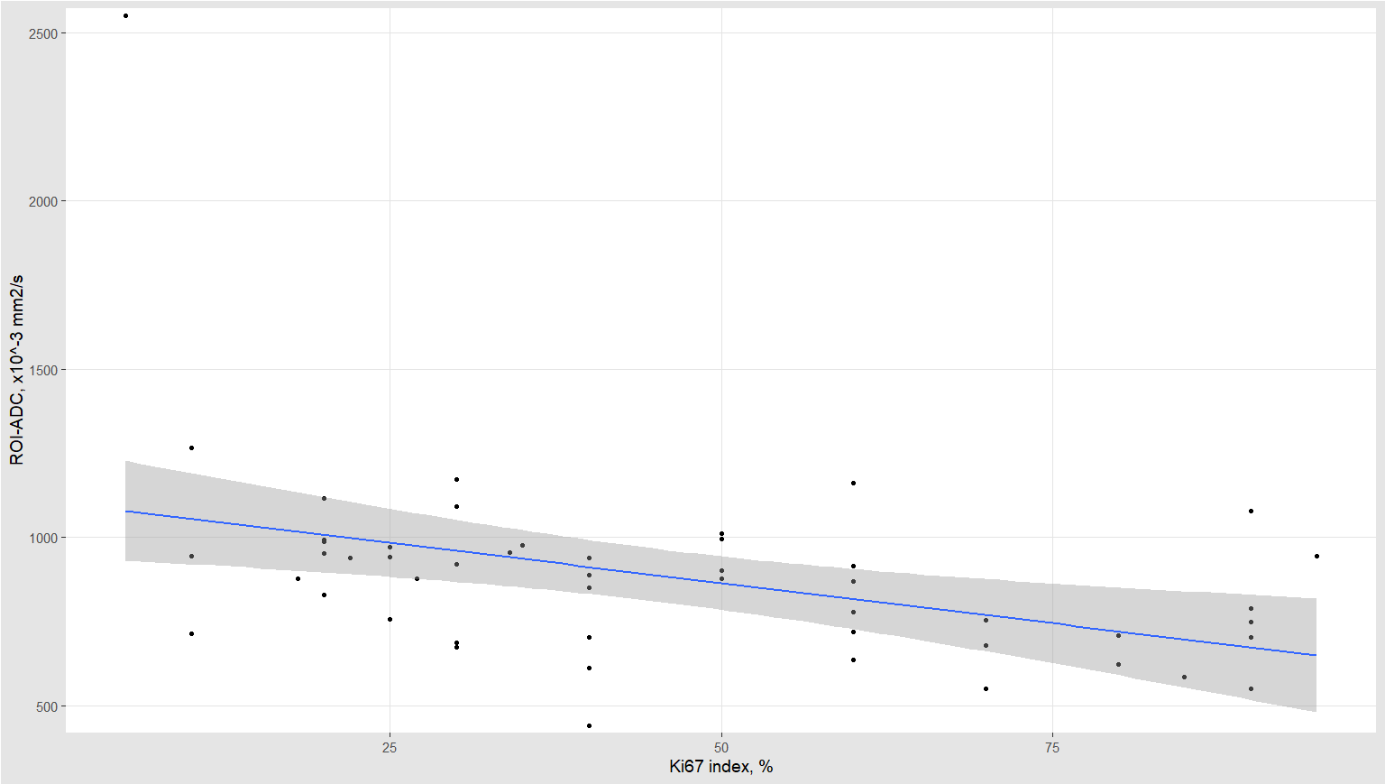
**

**Supplementary figure 1:** Scatter plot demonstrating the correlation between Ki67 proliferation index and region of interest (ROI)-apparent diffusion coefficient (ADC) before neoadjuvant chemotherapy.

**
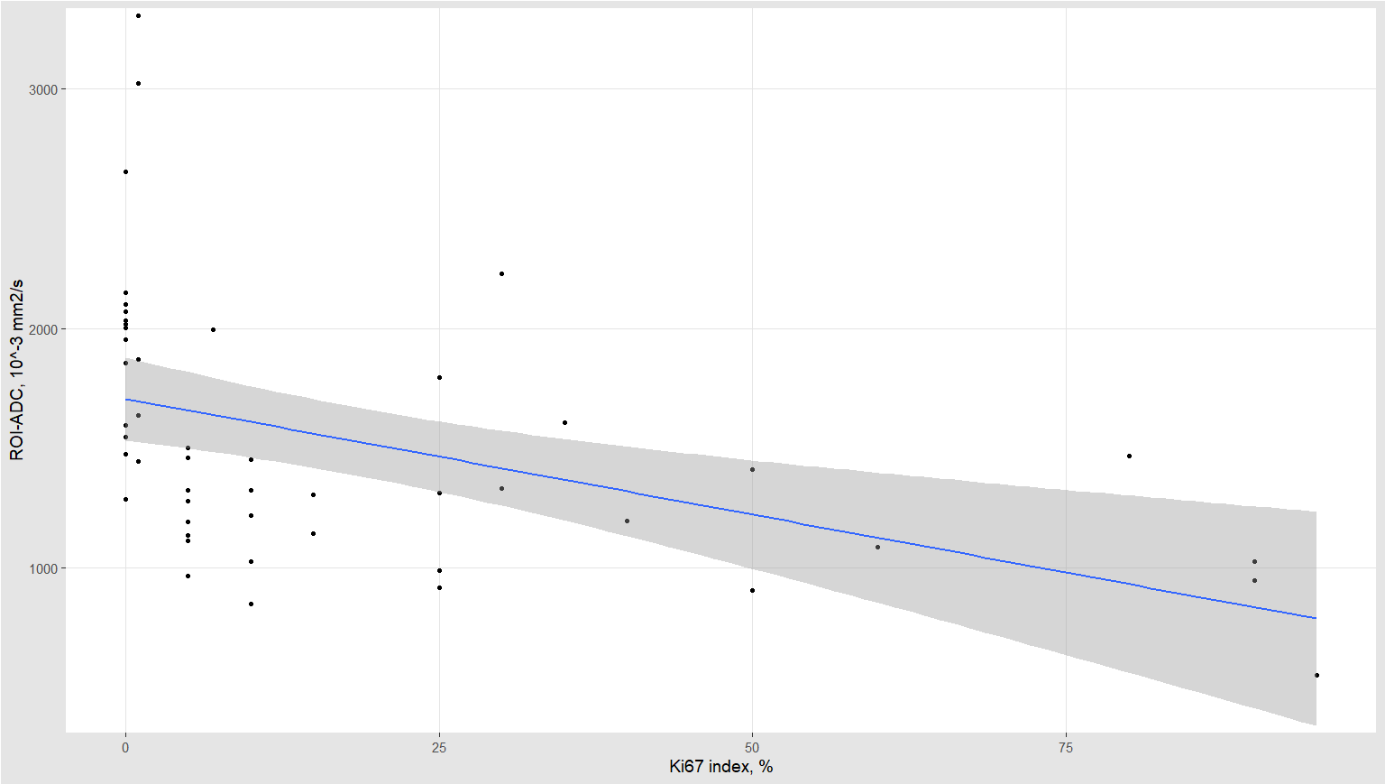
**

**Supplementary figure 2:** Scatter plot demonstrating the correlation between Ki67 proliferation index and region of interest (ROI)-apparent diffusion coefficient (ADC) after neoadjuvant chemotherapy.
